# *Peperomia pellucida*'s Ingredients, Antioxidant Properties, and Safe Usage as Food and Herbal Medicine

**DOI:** 10.4014/jmb.2406.06025

**Published:** 2024-09-20

**Authors:** Chau Thanh Tuan, Tran Thanh Men

**Affiliations:** 1 Institute of Food and Biotechnology, Can Tho University, Can Tho City 94000, Vietnam; 2 College of Natural Sciences, Can Tho University, Can Tho City 94000, Vietnam

**Keywords:** Antioxidants, *Drosophila melanogaster*, LD_50_, *Mus musculus*, *Peperomia pellucida*

## Abstract

*Peperomia pellucida* (L.) Kunth has traditionally been used as a vegetable and herbal medicine in certain countries, though its safety remains uncertain. In this study, we investigated the plant’s ingredients, volatile compounds, antioxidative activities, and toxicity in *Drosophila* and mice. The results revealed that *P. pellucida* contains volatile substances that give it a unique flavor, making it suitable for consumption and rich in antioxidant compounds, such as polyphenols, tannins, flavonoids, saponins, alkaloids, and terpenoids. The total phenolic content (TPC) and total flavonoid content (TFC) of the plant extract were 273.33 ± 4.91 mg gallic acid equivalents/g extract and 199.8 ± 0.346 mg quercetin equivalents/g extract, respectively. The remarkable antioxidative properties of the plant extract were demonstrated by nearly doubling the lifespan of *Drosophila* against oxidative stress. Moreover, the extract did not cause any acute or chronic toxicity in mice after being fed the plant, as indicated by the normal blood parameters and the absence of hepatic shape damage or impaired function. In light of these findings, *P. pellucida* is deemed safe for consumption and its bioactive ingredients can be extracted to create functional foods and pharmaceuticals.

## Introduction

*Peperomia pellucida* (L.) Kunth, a perennial plant and member of the *Piperaceae* family comprising 3,700 species, is widely cultivated in various regions globally, including South Asia, Madagascar, Africa, Australia, and New Zealand [1, 2]. This annual weed thrives in moist, loose soil and shady areas, preferring to grow during rainy periods [3]. Therefore, it commonly grows on large fruit trees. In many countries, *P. pellucida* is consumed as an herb or vegetable. Moreover, various solvents have been used to extract and identify its bioactive compounds.

*P. pellucida* is a widely recognized, natural remedy for alleviating a range of health issues, including inflammation, diabetes, dengue fever, and cardiovascular problems. It has even been used to stimulate hair growth [4-8]. Furthermore, its potential effects on cancer and the nervous system have also been investigated [9, 10]. This herbal remedy contains a plethora of bioactive compounds, including antioxidants, flavonoids, polyphenols, vitamins, essential oils, glycosides, and other volatile compounds [11-14]. Pellucidin, a compound with anti-inflammatory properties, has been identified in the plant [13, 15].

In Southeast Asian countries, such as Vietnam, *P. pellucida* (L.) Kunth is widely consumed raw as a vegetable or processed into salads as a mineral source [16]. Additionally, due to its unique flavor and taste, the plant is sometimes fermented or dried to make tea [17]. Several investigations have been carried out on the medicinal and bioactive activities of *P. pellucida* (L.) Kunth, and its antioxidative activity has been assessed [17].

Although the safety of *P. pellucida* for oral consumption in terms of food and medicine remains uncertain, it is essential to understand the acute and chronic toxicities in vivo and in vitro. Therefore, this study was conducted to examine the nutrient value of the plant, screen for antioxidative activities, particularly in vivo oxidative stress protection, and assess the acute and subchronic toxicities in flies and mice, respectively.

## Materials and Methods

Wild-type *Peperomia pellucida* (L.) Kunth was collected from Can Tho City (Vietnam). The collected plants were identified as members of the species in the laboratory. *Mus musculus* (Linnaeus) mice were supplied by the Pasteur Institute of Ho Chi Minh City and fed at the Laboratory of Animal Physiology, College of Education, Can Tho University. Canton S fruit flies (*Drosophila melanogaster*), provided by the Kyoto Institute of Technology, Japan, were maintained on standard medium under previously described conditions [5]. All chemicals used were of analytical grade.

### Extracting *Peperomia pellucida*

The collected samples were washed, and the damaged parts were removed, ground, and dried at 35°C using a cooling dryer. The dried samples were soaked in 96% ethanol at a ratio of 1/10 (m/v) for 24 h. The mixture was then filtered. The solution was collected and concentrated in vacuo using a rotary vacuum concentrator (R205 2 L, BUCHI Corp., Switzerland) to extract the compounds. Extracts were collected and stored at -18°C.

### Analysis of *P. pellucida* Compositions

Lipid concentration was determined using the Soxhlet method; protein was analyzed using the Kjeldahl method [18]; ash was determined using AACC [19], and moisture and available carbohydrates were analyzed using AOAC [20, 21]. Volatile compounds were identified using GC-MS at the Can Tho Technical Center of Standards Metrology and Quality (Vietnam). Other parameters were analyzed or determined following AOAC [22] or ISO protocols.

### Qualitative and Quantitative Screening for Antioxidative Activities of *P. pellucida* Extract

The compounds in *P. pellucida* extract were determined. Polyphenols, tannins, flavonoids, saponins, alkaloids, coumarins, quinines, and terpenoids were qualitatively determined using modified photochemical reactions [23].

The total flavonoid content of the extract was determined using quercetin as a standard [24]. Forty microliters of 5% Na_2_NO_2_ was mixed with a 400 μl solution containing 200 μl of extract and distilled water. After 5 min, 40 μl of 10% AlCl3 was added to the mixture, which was then incubated for 6 min. Then, 400 μl of 1 M NaOH was also added to the mixture, which was adjusted to 10 ml of distilled water. The absorbance of the mixture was measured at 510 nm wavelength using a V-730 spectrophotometer (JASCO Corp., Japan).

Total phenolic content was measured using the Folin-phenol method [25] with gallic acid as the standard. The extract, distilled water, and Folin-Ciocalteu were mixed (1:1:1, v/v). The mixture (250 μl) was incubated for 10 min. Then, 250 μl of 10% Na_2_CO_3_ was added, and the mixture was incubated at 40°C for 30 min. The absorbance of the solution was measured at 765 nm using a V-730 spectrophotometer (JASCO).

### In Vivo Assay in Drosophila for Antioxidative Activities

To investigate *Drosophila* resistance to oxidative stress, H_2_O_2_ was used. Male flies hatched within 48 h were selected and raised on a standard feed medium containing either 0.5 or 1 mg/ml of the extract for 10 days. After fasting for 2 h, the flies were placed in test vials with 10% H_2_O_2_ mixed with 9% glucose solution on blotting paper. The experiment was conducted in triplicate with 20 flies per group, as previously described [5, 26]. A positive control treatment was established using 0.05 mg/ml of gallic acid, while the control group received ordinary food. The number of surviving flies was recorded every 4 or 8 h and the initial decay time was calculated when more than 5% of the flies died. The 50% decay time (half-life) was calculated from the beginning of the experiment to the time at which 50% of the initial flies died.

### Scanning the Toxicity of *P. pellucida* Extract

**Determining acute toxicity.** Mice were randomly divided into groups of 10 (five males and five females in independent cages), starved for 24 h, and given free water. The mice in the control group freely drank water and ate feed; those in the exposed group (NT5000) were fed similarly to those in the control group, except that they were drunk with the *P. pellucida* ethanol extract at a dose of 5,000 mg/kg body weight at 11:00 a.m. They were then observed for live and active abilities, as well as convulsions, diarrhea, and foaming at the mouth after 1, 3, 6, 12, 24, 48, 72 h, and 7 days [27-29]. Afterward, they were anesthetized and operated on to collect their livers and 1 ml of blood from their hearts. The experiments were approved (coded: CTU-AEC24010) by the Animal Ethics Committee of Can Tho University.

**Determining subchronic toxicity.** Mice were divided and fed as in the acute toxicity experiment, except that they were starved for 18 h. The exposed mice (EX500) were drunk daily with *P. pellucida* ethanol extract at a dose of 500 mg/kg (0.2 ml/10 g each) of body weight [30] at 11:00 a.m. for 28 days, after which the mice were post-exposed for the next 30 days. They were then anesthetized and operated on as described previously.

### Data Analysis

Analysis of variance (ANOVA) was used to assess significant differences among multiple groups under various treatments, followed by Tukey's test using the Minitab 16 software (NSW, Australia) and GraphPad Prism (GraphPad Software, USA). In all groups, differences were considered statistically significant at *p* < 0.05. GraphPad Prism software was used for non-linear regression, processing, calculations, and graphs. Data are presented as mean ± SD of triplicate experiments.

## Results

### Characteristics of *P. pellucida*

**Fig. 1:**
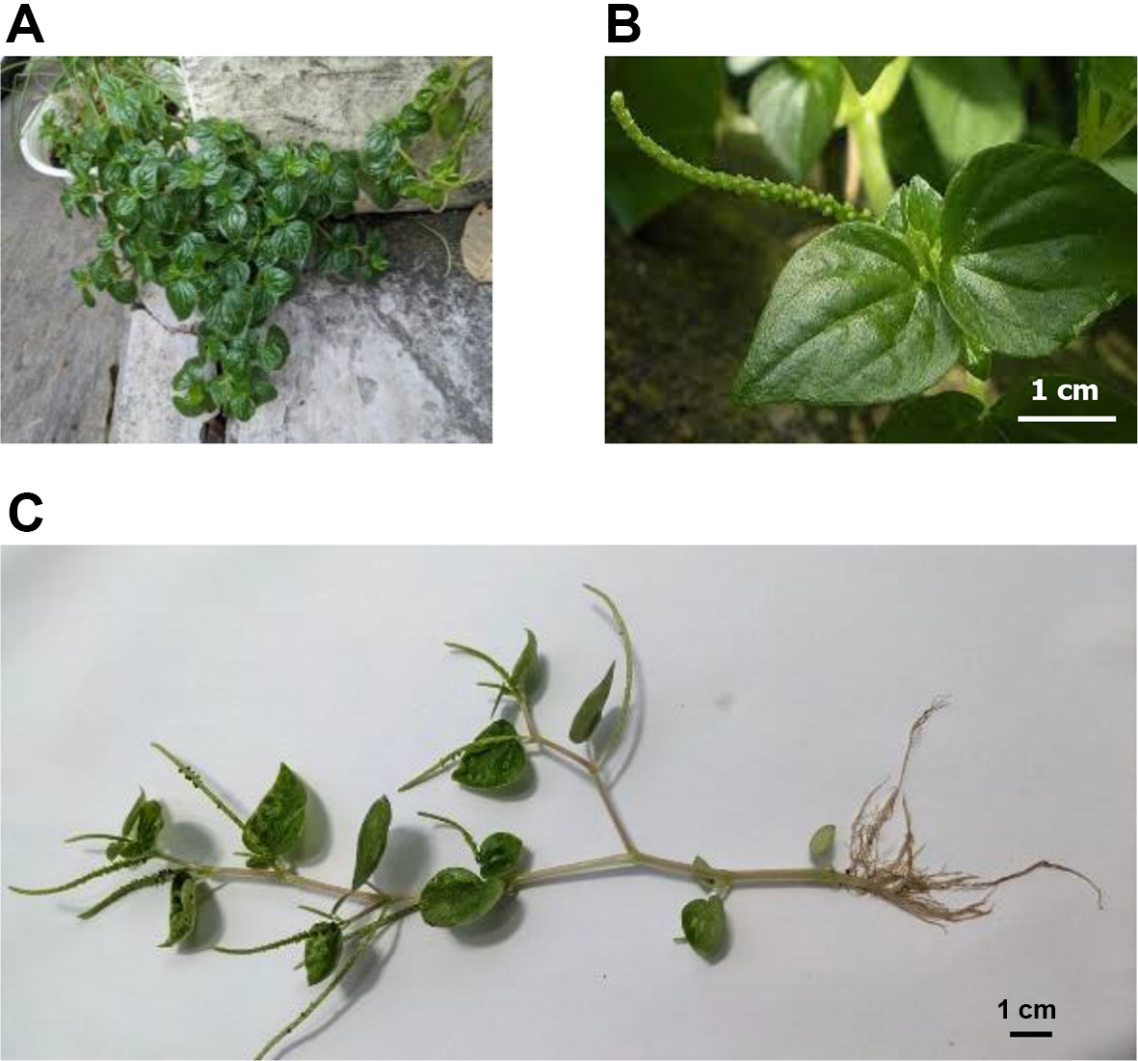
Botanical characteristics of *Peperomia pellucida* (L.) Kunth. (**A**) Wild-type *P. pellucida*. (**B**) Leaf shape of the vegetable. (**C**) The whole body of the vegetable. The trees were harvested in Can Tho City, Viet Nam. The collected samples were washed and damaged parts were removed and dried at room temperature and shade conditions.

Fig. 1 shows *P. pellucida* collected from Can Tho City home gardens growing abundantly on the ground. The stems were smooth, light green or white, 10–20 cm long, and 5 mm in diameter. The leaves were elliptical or heart-shaped, 1.5 to 3.5 cm long, with green upper and white or silver-green underneath sides, and had 5 to 7 propeller-shaped veins. The flowers were stemless, symmetrical, bisexual, cream-colored ovary forms, measuring 0.2 to 0.8 mm long. The seeds were black and 0.7 mm long.

The vegetable components included protein, carbohydrates, and a small amount of ash (Table 1). Additionally, Table 2 shows that the plant contains 17 volatile compounds, including esters, acids, and alcohols. The largest proportion, 37.62%, was 5-oxotetrahydrofuran-2,3-dicarboxylic acid, dimethyl ester, followed by dimethyl DL-malate (29.76%). Glutaric acid, 2-oxo-, dimethyl ester, 2-butenedioic acid (E)-, monomethyl ester, and citriconic anhydride comprised approximately 4.5%-9% of the three compounds. Six compounds comprised approximately 1-2% of the total, and the remaining compounds accounted for less than 1%.

### In Vitro and In Vivo Antioxidative Activities of *P. pellucida*

The antioxidative activities of *P. pellucida* were evaluated by confirming the presence of antioxidant compounds in the eight groups. The results in Table 3 indicate that the vegetable ethanol extract contained polyphenols, tannins, flavonoids, saponins, alkaloids, and terpenoids; coumarin and quinine were not detected. The total flavonoid and polyphenol content was quantitatively measured using quercetin and gallic acid, respectively. The antioxidant activity was determined to be 199.8 ± 0.346 mg quercetin equivalents (QE)/g extract and 273.33 ± 4.91 mg gallic acid equivalents (GAE)/g extract, as shown in Table 4.

**Fig. 2:**
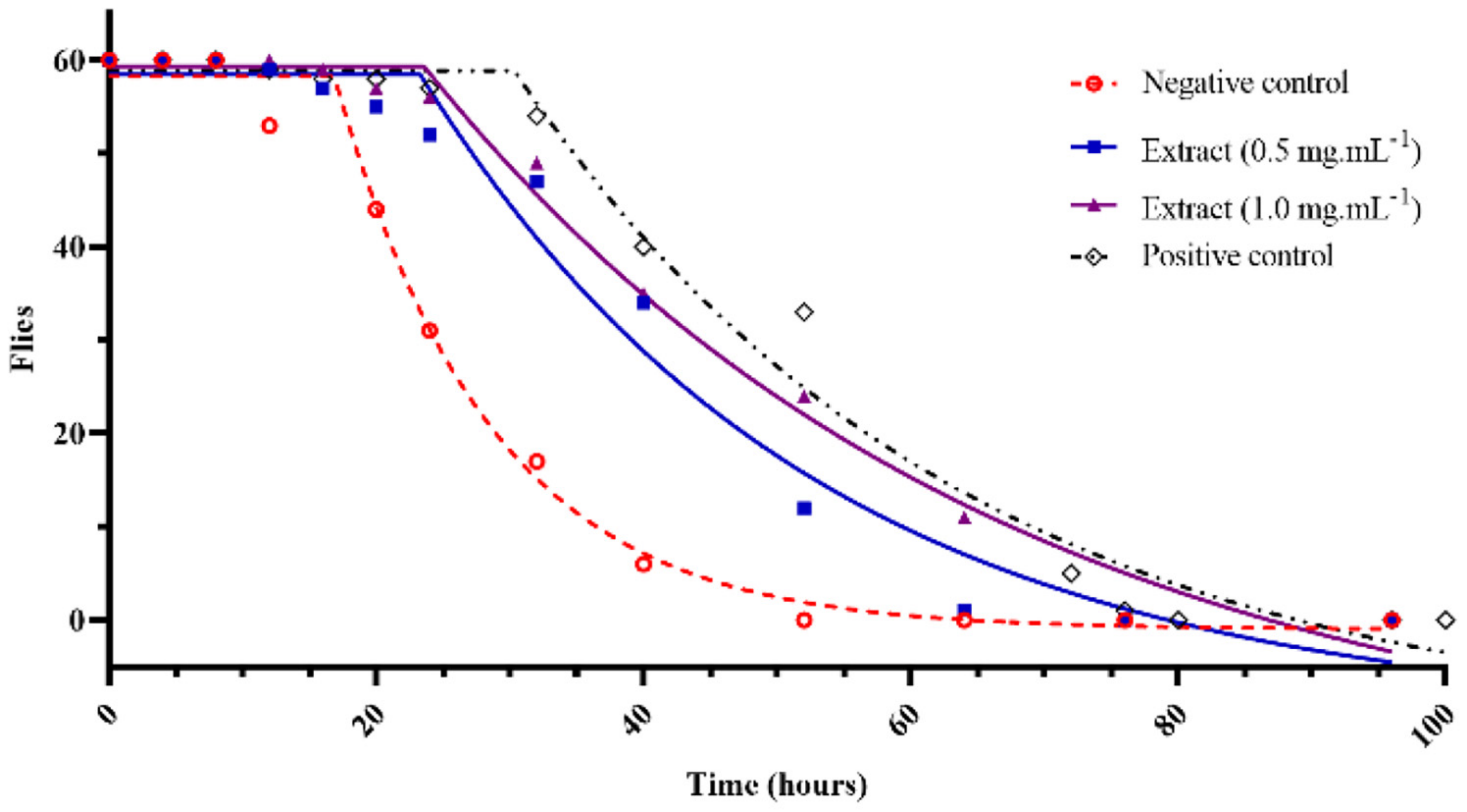
In vivo oxidative stress of flies by H_2_O_2_ shocking. Flies were placed in test vials with blotting paper containing 10% H_2_O_2_ mixed in 9% glucose solution and fed with normal medium (Negative control), 0.5 mg/ml and 1.0 mg/ml of the vegetable extract, and 0.05 mg/ml of gallic acid (Positive control). The flies were observed every 4-8 h during their life.

We conducted in vivo experiments in *Drosophila* using different concentrations of the extract and gallic acid as a positive control to assess antioxidative activity. The data were analyzed using non-linear regression, and as depicted in Fig. 2, the initial decay time for the 0.5 and 1.0 mg/ml extracts and gallic acid was 23.27 h, 23.57 h, and 30.34 h, respectively, which were significantly different from the negative control (16.86 h). Additionally, the half-life of the exposed flies was nearly double that of the negative control (42.22 ± 2.49 h and 47.69 ± 3.97 h, compared to 25.36 ± 1.49 h), and it was about 80% of the positive control (52.38 ± 4.99 h).

### Acute Toxicity of *P. pellucida*

This study demonstrated that the vegetable extract exhibited no acute toxicity in mice when administered at a dose of 5,000 mg/kg. Specifically, after 7 days of consumption, no mortality or unusual behavior, such as convulsions, ruffled hair, diarrhea, or vomiting, was observed in the mice (Table 5). Furthermore, the body and liver weights of the mice, as well as the proportion of the liver to the whole body, remained unchanged relative to the control group (Table 6). These findings suggest that the vegetable extract did not pose any acute toxicity risk to experimental mice.

Table 7 presents the hematological characteristics of mice, revealing that the levels of white blood cells (WBC), hemoglobin (HGB), mean corpuscular volume (MCV), neutrophils (NEU), lymphocytes (LYM), monocytes (MONO), eosinophils (EOS), and basophils (BASO) did not differ between the AT5000 mice and the control group. Additionally, liver and kidney function was evaluated using aspartate aminotransferase (AST), alanine aminotransferase (ALT), creatinine, and uric acid. The results indicated no significant differences between the contents of mice exposed to 500 mg extract/kg of body weight and those in the control group (*p* > 0.05). Furthermore, total serum protein content did not differ between the exposed and control mice.

**Fig. 3:**
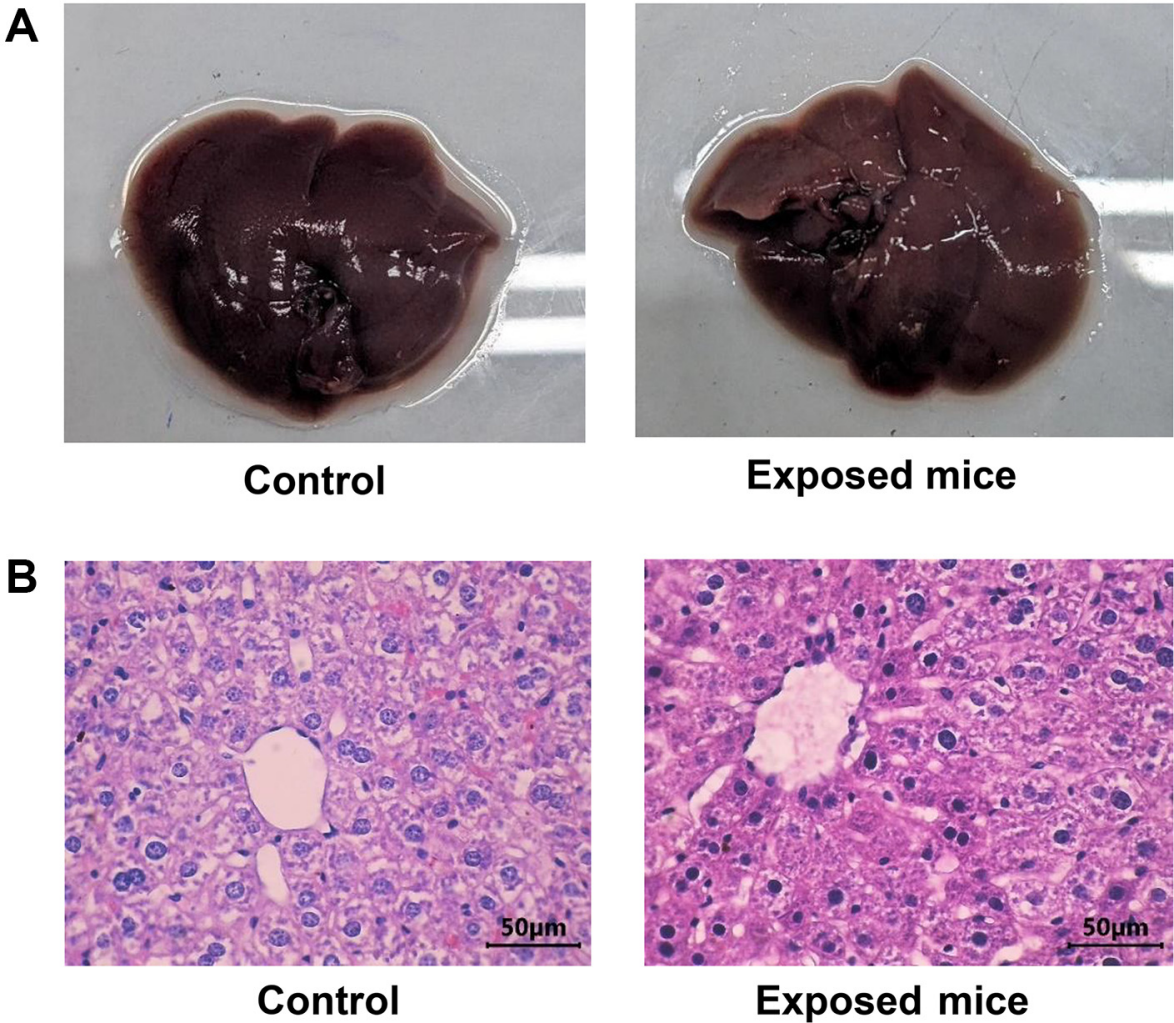
The mice's hepatic shape after acute toxic exposure. (**A**) The hepatic images were observed and captured under visible light in room conditions. (**B**) The images were observed under a 1000× microscope. Mice could freely drink and eat water and daily foods (Control); the exposed mice were fed similar to those of control except they drank the *Peperomia pellucida* (L.) Kunth ethanol extract in 10% DMSO with a dose of 5,000 mg/kg of body weight at 11:00 a.m for 7 days, observed after 1, 3, 6, 12, 24, 48, 72 h, and 7 days, generally anesthetized and operated on to collect their livers and 1 ml of blood from their hearts. The hepatic sample was immobilized by bouin and formaldehyde solutions, washed with ethanol, shaped by paraffin for 12 h, blocked, and cut into 4-5 μm thick layers, then triply soaked in toluene and ethanol.

Acute toxicity was assessed by examining the anatomy of mouse hepatic tissue. As depicted in Fig. 3, the cells in the central venous region of the livers of mice exposed to the extract were found to be in the normal stages. The hepatic cells were uniform in size, lacked edema or compression, and displayed spoke capillaries. Hepatocytes were arranged in radial rows, whereas phagocytes were observed outside central veins. Notably, no abnormalities, such as degeneration or swelling, were observed in the experiments, and there were no signs of bleeding in the anatomical structures.

### Subchronic Toxicity of *P. pellucida*

Chronic toxicity of plant extracts was evaluated over a period of 28 days. Mice that consumed a solution containing 500 mg of extract per kilogram of body weight did not exhibit any adverse reactions or symptoms. No mortality or unusual activities were observed and no clinical signs of toxicity were detected.

After 28 days of exposure, the body weights of the mice in the control group increased slightly compared with the initial measurements. However, after 14 days, the body weight of the control group did not change, and after 28 days, it decreased to 28.03 ± 2.48 g from the initial measurement of 31.83 ± 1.89 g (*p* < 0.05). In contrast, the exposed mice's body weight increased gradually from 29.57 ± 1.74 g to 34.52 ± 3.06 g (*p* < 0.05) over the period. The body weight of the exposed mice was significantly greater than that of the control group after 28 days of exposure (*p* < 0.05) (Table 8). Furthermore, no differences in hepatic weight were observed between mice exposed to 500 mg/kg extract and the control group.

Long-term trials in mice revealed that hematological parameters, including MCV, HGB, HCT, MCH, and MCHC, remained unchanged when exposed to the extract, similar to the outcomes of the acute toxicity tests. However, as shown in Table 9, a statistically significant decrease was observed in the RBC count (7.88 ± 0.87 10^6^/ml) compared to the control group (9.71 ± 0.58 10^6^/ml) (*p* < 0.05). Moreover, the mean concentrations of AST and ALT were 107.8 ± 22.5 and 58.0 ± 6.96, respectively, as shown in Table 9, indicating that there was no significant difference in the levels of hepatic enzymes, specifically AST and ALT, between mice exposed to 500 mg of extract and those that were not. Furthermore, no notable changes were observed in kidney function, as evidenced by the comparable levels of creatinine and uric acid between the two groups.

**Fig. 4:**
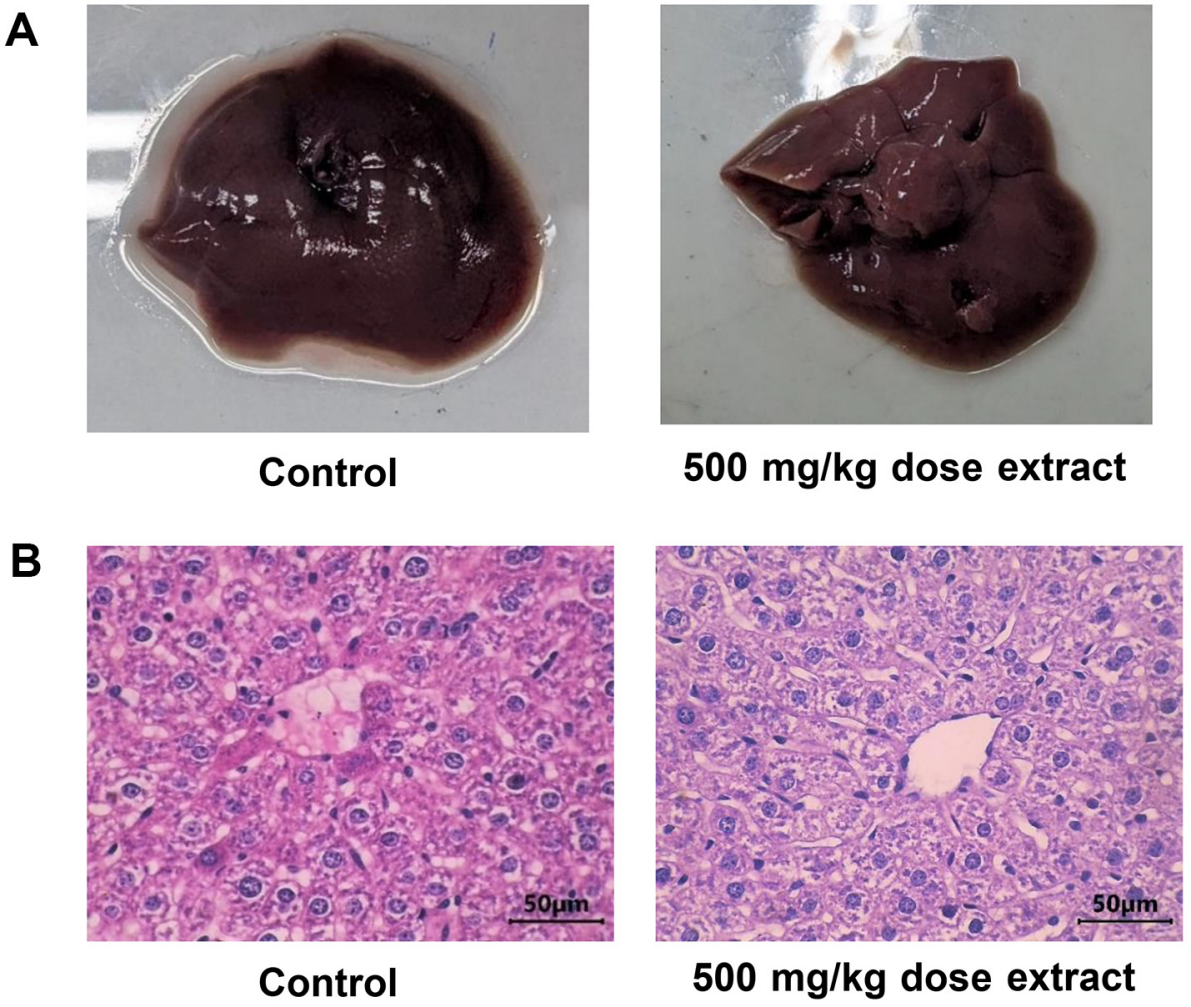
The mice's hepatic images after chronic toxic exposure. (**A**) Visible light in room conditions. (**B**) The 1000× microscopic images. The hepatic images were observed and captured as described in the legend of Fig. 3. Mice could freely drink and eat water and daily foods (Control); the exposed mice were fed similar to those of control except they were drunk the *Peperomia pellucida* (L.) Kunth ethanol extract in 10% DMSO with a daily dose of 500 mg/kg at noon. The hepatic sample was treated as described in the legends of Fig. 3.

Fig. 4 illustrates the hepatic tissues of mice exposed to 500 mg/kg. The cells were regular stage, high-density endoplasmic, and fully covered; the hepatocytes were not swollen, arranged in radial rows, and revealed spoke capillaries. Phagocytes outside the central veins, degeneration or swelling of the nucleus cells, and tissue bleeding were not observed. These characteristics were similar to those observed in the control group (Fig. 4B).

The hepatic tissues of mice exposed to 500 mg/kg are shown in Fig. 4. The cells in this tissue were at a regular stage, with high-density endoplasmic reticulum and fully covered hepatocytes that were not swollen and were arranged in radial rows. Additionally, the tissue exhibited spoke capillaries. It is important to note that phagocytes were not found outside the central veins and there was no degeneration, swelling of the nucleus cells, or tissue bleeding. These characteristics are consistent with those observed in the control group (Fig. 4B).

## Discussion

The morphological attributes of *P. pellucida* analyzed in this study were consistent with those previously reported by other authors [31]. This finding indicates that the investigated plant species is similar to the one previously described, is widely distributed, and can be readily found in tropical and subtropical regions such as Southeast Asia, South America, Africa, and Australia [1, 2]. The 17 volatile compounds (Table 2) identified by GS-MS can be categorized into four groups: ester, acid, alcohol, and anhydride, with ester compounds being the most abundant. These ester compounds can interact with the human olfactory system and are responsible for the perception of food flavors. For instance, malate compounds contribute to a fruity odor, whereas anhydrides are found in Arabica coffee [32]. Additionally, the presence of saponins, alkaloids, and fifty oils [14] in *P. pellucida* can influence consumers’ sensory experience. Consequently, these compounds contribute to the unique flavor and taste of the plant.

*P. pellucida* possesses various bio-activity compounds [3, 5] and exhibits anti-inflammatory properties [15, 17, 33]. The in vivo effects of carrageenin and arachidonic acid on prostaglandin synthesis in rats and mice [34] have demonstrated their algesic properties. *P. pellucida* exhibits anti-inflammatory activity related to the immune response by inhibiting cylooxygenase-1 [35] and cylooxygenase-2 [33]. The initial decay times of *Drosophila*, in vivo, displayed a considerable distinction between the negative control and treatment groups (Fig. 2), indicating that the *P. pellucida* extract could safeguard flies against oxidative stress more effectively than those without the plant extract. Furthermore, the half-life of the exposed flies was nearly double that of the negative control and approximately 80% that of the positive control, unequivocally demonstrating protection. The anti-stress capacity of the plant is attributable to the internal varieties of antioxidative compounds [7] (Table 3) and the high concentrations of TPC and TFC (Table 4).

Subchronic toxicity and changes in body weight are two essential parameters that can be used to assess the side effects of drugs and chemicals [36]. The results of a study that administered a maximum dose of 5,000 mg/kg for 7 days and 500 mg/kg for 28 days (Table 5) showed that the LD_50_ value of *P. pellucida* extract was higher than that of the administered dose, suggesting that the extract is safe for oral consumption. It is crucial to determine an animal's physiological and pathological state in animal research, and one of the critical factors is the weight of the animal's organs, which can indicate whether an organ has been damaged or affected by toxins [37]. In this study, subchronic toxicity observations at a dose of 500 mg/kg for 28 days (Table 9) showed no significant difference in hepatic and body weights between mice exposed to the extract and the control group (Tables 6 and 8). The consistency in the percentage of liver weight and body weight suggests that the extract had no adverse effects on the growth and development of the mice or their hepatic weight, indicating that the plant was not chronically toxic to the animal. This finding is consistent with that of an experiment conducted using a Brazilian plant [34].

Assessing the effects of toxins on various tissues, particularly the liver and kidneys, is crucial in toxicological research [38-40]. These two organs play a vital role in the survival of an organism because they are responsible for detoxification and the maintenance of homeostasis [41]. To evaluate the potential toxicity, it is essential to examine the shape and function of the liver, which is primarily responsible for chemical metabolism [36]. The hepatic shapes of the animals did not change after exposure to the extracts in acute and chronic experiments (Figs. 3 and 4). The most commonly used indicators of liver damage, AST and ALT [42], were unaffected by the extract. Similarly, kidney function is evaluated by measuring blood urea and creatinine levels, as the kidneys are responsible for reabsorbing essential substances and removing waste products [43].

AST (SGOT) and ALT (SGPT) enzyme levels are commonly used to evaluate liver diseases, such as acute and chronic hepatitis, as well as liver parenchymal damage caused by viral and alcoholic hepatitis. In this study, we assessed the effect of 5,000 mg/kg *P. pellucida* extract on AST and ALT levels in mice, in comparison to control mice, to determine whether the extract caused any liver damage. The results indicated that the extract did not increase the AST and ALT indices in mice, suggesting that it did not cause liver damage. This finding aligns with the hepatic enzyme index assessment, demonstrating that a dose of 5,000 mg/kg *P. pellucida* extract does not have an impact on the liver.

Assessment of liver and kidney function included evaluation of creatinine, AST, ALT, uric acid, and serum total protein. The results demonstrated that serum creatinine is a byproduct of creatinine phosphate breakdown in muscle tissue, which is entirely filtered through the glomeruli in the kidneys and is not reabsorbed by the renal tubules. Elevated uric acid levels may indicate kidney damage, kidney failure, or reduced uric acid production due to liver cell damage. However, recent studies in mice suggest that the administration of *P. pellucida* extract does not significantly affect uric acid levels compared to the control group. Based on these findings, it can be inferred that the *P. pellucida* extract does not induce acute nephrotoxicity and is compatible with a previous study showing that the plant exerts activities that can help in treating kidney diseases [35].

The hematological index is an exceptionally sensitive marker for toxic substances and serves as a critical indicator of physiological and pathological states in both humans and animals [44]. Assessing blood parameters is of paramount importance when evaluating toxicity, and alterations in hematological values possess substantial predictive potential for toxicity in humans [45]. The hematopoietic system, responsible for the production of red blood cells, is particularly susceptible to harm from toxic chemicals, especially in the bone marrow [46]. Consequently, prolonged use of the *P. pellucida* extract can affect the hematopoietic system, resulting in a decreased red blood cell count. It is crucial to consider this when employing the plant for extended periods of treatment.

## Conclusion

*P. pellucida* contains volatile constituents that lend it a unique flavor and composition, as well as ingredients suitable for culinary use. This vegetable contains a range of antioxidant compounds, such as polyphenols, tannins, flavonoids, saponins, alkaloids, and terpenoids. Interestingly, coumarin and quinine were not detected in the plant. *P. pellucida*'s TPC and TFC are 199.8 ± 0.346 mg QE/g extract and 273.33 ± 4.91 mg GAE/g extract, respectively. In addition, the plant demonstrates unambiguous antioxidative properties and extended the lifespan of *Drosophila* against oxidative stress. These activities can almost double the half-life of the flies, from 25.36 ± 1.49 h to 42.22 ± 2.49 h and 47.69 ± 3.97 h. The plant extract was non-toxic to mice and caused neither acute nor chronic harm. It did not cause hepatic shape damage or impaired function, and the blood parameters were within normal limits. Therefore, *P. pellucida* is suitable for use in food, and can also be used to develop functional foods and pharmaceuticals.

## Figures and Tables

**Table 1:** Proximate compositions of *Peperomia pellucida* (L.) Kunth from the Mekong Delta.

Ingredients	Value (% wt)
Moisture	95.58 ± 0.02
Ash	0.84 ± 0.01
Protein	1.22 ± 0.02
Lipid	ND
Carbohydrate	2.36 ± 0.05
Fiber	1.06 ± 0.03
Available carbohydrate	1.30 ± 0.08

ND: Non detective, LOD < 0.3

**Table 2:** Volatile compounds of *P. pellucida* 96% ethanol extract analyzed by GC-MS.

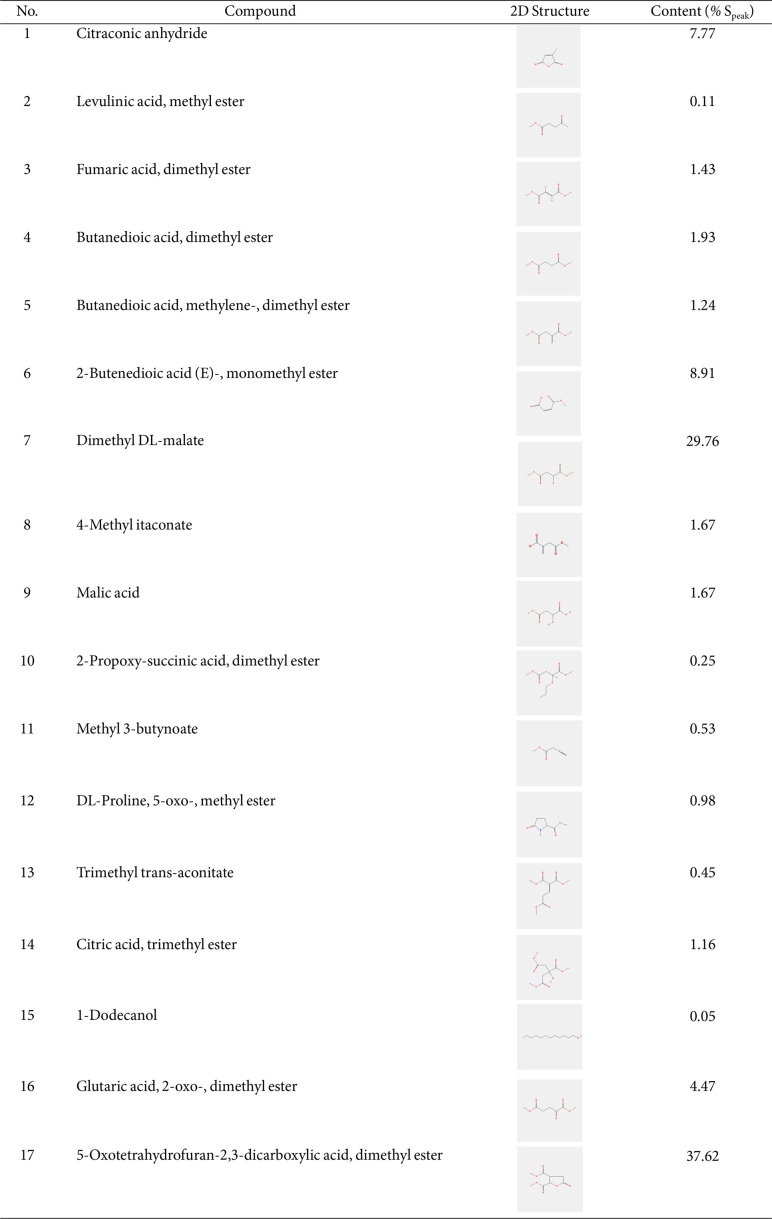

**Table 3:** Qualitative determination of chemical compound in *P. pellucida* 96% ethanol extract.

Compound	Reagent	Indicator	Result
Polyphenol	FeCl_3_ 10%	Changed to blue or violet blue solution	+
Tannin	Gelatin 1%	White precipitate Appeared	+
Flavonoid	FeCl_3_ 5%	Changed to black solution	+
Saponin	Ethanol	Appeared foams in solution	+
Alkaloid	Wagner	Appeared slightly yellow to brown precipitate	+
Coumarin	NaOH 10%	Changed to yellow solution	-
Quinone	Saturated HCl	Changed to green solution	-
Terpenoids	Saturated CHCl_3_ + Saturated H_2_SO_4_	Changed to green or red solution	+

219.62 g dried *P. pellucida* was extracted with 96% ethanol and collected the extract with a yield of 7.32%. (+) the compound existed, (-) the compound did not exist.

**Table 4:** Contents of total flavonoids and polyphenols in *P. pellucida* 96% ethanol extract.

Compound	Standardized equation	Content
Total flavonoid	y = 0.0047x + 0.0192 (R^2^ = 0.9896)	199.8 ± 0.346 mg quercetin equivalents /g extract
Total polyphenol	y = 0.0298x – 0.2692 (R^2^ = 0.9904)	273.33 ± 4.91 mg gallic acid equivalents/g extract

**Table 5:** Quantity of dead mice within 7 days of drinking in *P. pellucida* 96% ethanol extract in the acute toxic experiments.

Group	Mice (Individual)	Body weight (g)	Drinking amount (ml, 3 times)	Live mice/Dead mice (Individual)	
				Within 1 day	Within 7 days
Control	10	31.89 ± 2.45^a^	0.9	10/0	10/0
NT5000	10	29.50 ± 5.12^a^	0.9	10/0	10/0

NT5000: mice exposed to 5000 mg extract/kg of body weight; Control; mice fed with normal conditions.

**Table 6:** Body and hepatic weight of mice before and after drinking the extract in the acute toxic experiments.

Group	Body weight (g)		HW/BW After 7 days (%)
	Initial day	7 day	
Control	31.89 ± 2.45^a^	34.33 ± 2.38^a^	5.667 ± 0.627^a^
NT5000	29.50 ± 5.12^a^	31.37 ± 5.68^a^	5.781 ± 1.402^a^

NT5000: mice exposed to 5000 mg extract/kg of body weight; Control; mice fed with normal conditions. HW/BW: Percentage of mouse hepatic and body weight. Different letters of average and error values in a row indicated the statistical mean of significant difference (*p* < 0.05).

**Table 7:** Effects of *P. pellucida* extract on hematological and biochemical parameters in mouse acute toxic experiments.

Parameter	Unit	Group	
		Control	NT5000
Hematological parameters			
WBC	10^3^/μl	3.122 ± 0.966^a^	3.776 ± 1.165^a^
NEU	10^3^/μl	0.686 ± 0.275^a^	1.086 ± 0.677^a^
LYM	10^3^/μl	2.241 ± 0.870^a^	2.574 ± 0.984^a^
MONO	g/l	0.151 ± 0.217^a^	0.042 ± 0.061^a^
EOS	g/l	0.004 ± 0.004^a^	0.004 ± 0.002^a^
BASO	g/l	0.042 ± 0.028^a^	0.071 ± 0.071^a^
HGB	g/dl	14.200 ± 1.131^a^	13.880 ± 1.098^a^
MCV	μm^3^	52.200 ± 1.834^a^	51.300 ± 2.240^a^
Biochemical parameters			
Creatinine	μmol/l	62.40 ± 3.85^a^	64.00 ± 0.46^a^
AST	U/l	62.60 ± 3.44^a^	104.20 ± 10.9^a^
ALT	U/l	24.40 ± 4.56^a^	65.60 ± 45.0^a^
Acid Uric	μmol/l	230.60 ± 64.7^a^	189.20 ± 13.9^a^
Protein T. P	g/l	54.00 ± 6.04^a^	52.80 ± 7.40^a^

WBC: White body cell, HBG: Hemoglobin, MCV: Mean corpuscular volume, NEU: Neutrophil, LYM: Lymphocyte, MONO: Monocyte, EOS: Eosinophil, BASO: Basophil, AST: Aspartate aminotransferase, and ALT: Alanine aminotransferase. Different letters of average and error values in a row indicated the statistical mean of significant difference (*p* < 0.05).

**Table 8:** Body and hepatic weight of mice before and after drinking the extract in the chronic toxic experiments.

Group	Body weight (g)			Hepatic weight	
	Initial day	7 days	28 days	28 days (g)	HW/BW after 28 days (%)
Control	31.83 ± 1.89^bA^	32.71 ± 1.47^bA^	28.03 ± 2.48^aA^	1.27 ± 0.42^A^	4.38 ± 1.04^A^
EX500	29.57 ± 1.74^aA^	31.91 ± 2.51^abA^	34.52 ± 3.06^bB^	1.96 ± 0.30^A^	5.71 ± 0.95^A^

EX500: mice exposed to 500 mg extract/kg of body weight; Control; mice fed with normal conditions. HW/BW: Percentage of mouse hepatic and body weight. Different letters (Row: uppercase letter, Column: lowercase letter) of average and error values in a row indicated the statistical mean of significant difference (*p* < 0.05).

**Table 9:** Effects of 500 mg/kg dose of *P. pellucida* extract on hematological and biochemical parameters in mouse chronic toxic experiments.

Parameter	Unit	Control	EX500
Hematological parameters			
WBC	10^3^/μl	4.55 ± 1.01^a^	4.43 ± 0.96^a^
HGB	g/dl	14.20 ± 0.97^a^	12.14 ± 1.36^a^
MCV	μm^3^	55.76 ± 1.86^a^	57.30 ± 0.84^a^
RBC	10^6^/μl	9.71 ± 0.58^a^	7.88 ± 0.87^b^
HCT	%	54.16 ± 4.25^a^	45.16 ± 4.89^a^
MCH	pg	14.64 ± 0.74^a^	15.40 ± 0.14^a^
MCHC	g/dl	26.26 ± 1.05^a^	26.90 ± 0.39^a^
Biochemical parameters			
Creatinine	μmol/l	266.6 ± 60.4^a^	332.0 ± 20.47^a^
AST	U/l	100.2 ± 34.3^a^	107.8 ± 22.5^a^
ALT	U/l	51.2 ± 18.43^a^	58.0 ± 6.96^a^
Acid Uric	μmol/l	302.0 ± 46.0^a^	326.0 ± 54.6^a^

EX500: mice exposed to 500 mg extract/kg of body weight; Control; mice fed with normal conditions. WBC: White body cell, HGB: Hemoglobin, MCV: Mean Corpuscular Volume, RBC: Red Blood Cell, HCT: Hematocrit, MCH: Mean Corpuscular Hemoglobin, MCHC: Mean Corpuscular Hemoglobin Concentration. AST, and ALT were expressed in note of Table 7. Different letters of average and error values in a row indicated the statistical mean of significant difference (*p* < 0.05).
